# Coumestrol facilitates apoptosis in colorectal cancer cells by interacting with ZIP8 protein via the ferroptosis pathway

**DOI:** 10.7150/jca.94628

**Published:** 2024-07-02

**Authors:** Jing Geng, Yingying Wang, Fengchun Lv, Xiaomin Yu, Mingyu Gong, Jie Zhang, Zicheng Zhao, Xiaoyue Zhu, Xiaoyu Zhang, Jian Yang, Xiu-An Yang

**Affiliations:** 1 Laboratory of Gene Engineering and Genomics, School of Basic Medical Sciences, Chengde Medical University, 067000 Chengde, China.; 2 Graduate School of Chengde Medical University, 067000 Chengde, China.; 3 Department of Biomedical Engineering, Chengde Medical University, 067000 Chengde, China.; 4 Institute of Basic Medical Sciences, School of Basic Medical Sciences, Chengde Medical University, 067000 Chengde, China.; 5 Hebei Key Laboratory of Nerve Injury and Repair, Chengde Medical University, 067000 Chengde, China.

**Keywords:** colorectal cancer, coumestrol, SLC39A8, ZIP8, ferroptosis pathway

## Abstract

**Objective:** So far, there have been no reports of coumestrol inhibiting colorectal cancer (CRC) through the ferroptosis pathway. This study is to investigate the mechanism of the traditional Chinese medicine monomer coumestrol in the treatment of CRC.

**Methods:** Data on CRC transcriptome sequencing was obtained from the GEO database and TCGA database. Bioinformatics analyses were conducted to screen for CRC prognostic-related key genes and their potential binding monomers in traditional Chinese medicine. The inhibitory effect of coumestrol on CRC cell lines (COLO 205 & HCT 116) was determined using the CCK-8 assay, and cell apoptosis was assessed by flow cytometry. The content of ferrous ions was measured using the Ferrous Ion Content Assay Kit. The expression of ferroptosis pathway-related genes SLC39A8, NCOA4, VDAC2, and NOX2 before and after small interference RNA (siRNA) was examined through real-time PCR and Western blotting.

**Results:** SLC39A8 was found to be associated with CRC clinical progression staging, and its encoded protein ZIP8 may bind to coumestrol. KEGG enrichment analysis suggested that ZIP8 plays a role in iron transmembrane transport and may affect the expression of ferroptosis pathway-related genes NCOA4, VDAC2, and NOX2. Coumestrol was found to induce apoptosis in CRC cell lines by upregulating the expression of ferroptosis pathway-related genes SLC39A8, NCOA4, VDAC2, and NOX2. However, coumestrol was unable to upregulate the expression of ferroptosis pathway-related genes in CRC cell lines after SLC39A8 interference.

**Conclusion:** Coumestrol facilitates apoptosis in CRC cells by interacting with ZIP8 protein via the ferroptosis pathway.

## Introduction

Colorectal cancer (CRC) is one of the most prevalent cancers globally, ranking third for incidence and second for mortality. CRC accounts for approximately 80,000 new cases and 8.81 million deaths each year 1. Currently, surgery remains the primary approach for treating early-stage CRC (stage I and II), with a 5-year survival rate of approximately 90% 2. Therefore, early screening and diagnosis play a pivotal role in effectively managing CRC patients. Unfortunately, there is still a lack of suitable methods for early CRC diagnosis. Inflammatory markers, such as fecal calprotectin (FC) and chitinase protein (CHI3L1), have the potential to serve as biomarkers for early CRC diagnosis 3, 4. However, these widely recognized markers display variability in terms of their sensitivity and specificity, emphasizing the necessity for new biomarkers in early CRC screening.

Ferroptosis, a recently identified regulated cell death pathway, is characterized by the accumulation of iron, which is dependent on lipids. This accumulation of iron raises levels of lethal reactive oxygen species 5. Additionally, the excessive presence of iron within cells triggers lipid peroxidation, leading to cell death and resulting in distinct morphological and metabolic changes 6. Therefore, activation of ferroptosis holds the potential to hinder the proliferation of tumor cells, offering a promising avenue for cancer treatment through the targeting of ferroptosis in these cells 7.

The active ingredient in traditional Chinese medicine are the monomers, which serve as the foundation for its mechanism of action. The monomers have the potential to play a crucial role in the anti-tumor properties. There is well-documented evidence that coumarin can inhibit the proliferation and metastasis of human CRC HCT 116 cells through the Wnt signal 3. Additionally, coumarin has the ability to induce endoplasmic reticulum stress and promote apoptosis in human CRC HT 29 cells 8. Coumestrol, a crystalline compound possessing estrogenic activity and a derivative of coumarin, may therefore have the potential to serve as a crucial monomer for anti-tumor properties.

In recent years, the combination of genetic sequencing technology and bioinformatics tools has played a significant role in the discovery of tumor biomarkers. Databases such as The Cancer Genome Atlas (TCGA) and Gene Expression Omnibus (GEO) are commonly used to screen differentially expressed genes related to CRC 9-11. In this study, we obtained CRC RNA sequencing data from the TCGA and GEO databases to identify key genes associated with the tumor. Subsequently, we conducted a screening of traditional Chinese medicine monomers to determine their effects on these tumor-associated key genes. We further investigated the effects of these monomers on two CRC cell lines.

## Materials and methods

### Data resources

Sample information, including RNA expression data, clinical data, and experimental data of CRC, was downloaded from the GEO (https://www.ncbi.nlm.nih.gov/gds/?term=) database and TCGA (https://www.cancer.gov/ccg/research/genome-sequencing/tcga) database. In total, three datasets were downloaded, consisting of two GEO datasets and one TCGA dataset, with a combined total of 1,343 samples. Out of these samples, 60 were normal samples, while 1,283 were tumor samples. Specifically, the GSE39582, GSE17538, and TCGA-COAD datasets contained 19/566, 0/244, and 41/473 samples in the normal and tumor groups, respectively.

### Identification of key genes associated with CRC progression

A univariate Cox proportional hazard regression analysis was initially conducted using the "survival" package to validate the association between individual genes and patients' overall survival (OS). Clinical information, including age, sex, and stage, was integrated with gene expression levels to estimate the correlation between clinical features and gene expression. Then, single-factor independent prognostic analysis was performed utilizing the "survival" R package. To identify genes with differential expression between normal and tumor tissues within the same patient, paired difference analysis was performed for TCGA dataset using the R packages "limma" and "ggpubr". Finally, Kaplan-Meier survival analysis was implemented using the "survival" and "survminer" R packages to compare the OS of patients based on key gene expression levels.

### Traditional Chinese medicine monomer screening

The monomers that can potentially bind with tumor-associated key genes in Traditional Chinese medicine were screened from BenCaoZuJian (http://herb.ac.cn/) and TCMSP database (https://old.tcmsp-e.com/tcmsp.php). The screening criteria were set as OB ≥ 30% and DL ≥ 0.18. The 2D structure of the monomer was downloaded from PubChem (https://pubchem.ncbi.nlm.nih.gov/) and then converted to a 3D model using ChemBio3D Ultra 14.0, with the optimization model set at the minimum free energy. The 3D model of the tumor-associated key gene was obtained from the UniProt website (https://www.uniprot.org) and processed using PyMOL 2.4.0a0 (https://pymol.org/2/) to remove water molecules and other ligands. Subsequently, the model was hydrogenated and saved as a PDBQT file with the assistance of AutoDockTools 1.5.6 (https://autodock.scripps.edu/). This step aimed to determine the active pocket and identify the site for small molecule docking within the defined scope. Molecular docking was performed using AutoDock Vina 1.1.2 (https://vina.scripps.edu/). Finally, structure visualization was conducted using PyMOL software (http://www.pymol.org/).

### Gene ontology (GO) and Kyoto Encyclopedia of Genes and Genomes (KEGG) functional enrichment analysis

GO and KEGG biological functional pathway enrichment analyses were performed for the identified tumor associated key gene using the “clusterProfiler” R package 12. The cutoff criterion was defined as FDR <0.05 and |log2FC| ≥1.

### Cell culture

The human normal colon immortalized epithelial cell line NCM460 was purchased from Shanghai Gaining Biotechnology Co., Ltd. The human colorectal adenocarcinoma cell (COLO 205) and human colon cancer cell (HCT 116) were both provided by Hunan Fenghui Biotechnology Co., Ltd. Coumestrol (#B29170) was purchased from Shanghai Yuanye Biotechnology Co., Ltd. All cell lines were grown at 37 ℃ and 5% CO_2_ in Roswell Park Memorial Institute (RPMI)-1640 medium (Procell Life Science&Technology Co.,Ltd., Wuhan, China) supplemented with 10% heat-inactivated fetal bovine serum (FBS; Thermo Fisher Scientific, Inc.,Waltham, USA), 100 μg/mL streptomycin, and 100 U/mL of penicillin.

### CCK-8

The Cell Counting Kit-8 (CCK-8) (#K1018, APExBIO) was used for the coumestrol inhibition rate assay experiment on NCM460 and CRC cell lines. A CCK-8 working solution was prepared in a dark centrifuge tube, with a ratio of CCK-8 to basic culture medium of 1:9. A volume of 100 µL of the CCK-8 working solution was added to each well, including a blank control which only contained the CCK-8 working solution. After incubating for 4 hours at 37 ℃ in a CO_2_ incubator, the OD value was detected at 450 nm using a Microplate Reader (#HBS-1096C, DeTie, Nanjing). The cell inhibition rate (%) was calculated using the formula [(Ac-As)/(Ac-Ab)]×100%. "Ac" represents the absorbance of the control well, which contained cells, culture medium, and CCK-8. "As" represents the absorbance of the experimental wells, which included cells, culture medium, CCK-8, and the compound being tested. "Ab" represents the absorbance of the blank wells, which contained only culture medium and CCK-8. Three independent experiments were conducted to overcome random error.

### Ki67 immunofluorescence staining

After treatment with coumestrol (100 µM) for 96 hours, COLO 205 and HCT 116 cells were fixed with 4% paraformaldehyde in PBS for 20 minutes, washed with PBS three times, and then permeabilized in 0.1% Triton X-100 for 20 minutes. Subsequently, after blocking in 3% donkey serum for an hour, the primary antibody Ki67 (#A16919, ABclonal, China) was incubated overnight at 4 ℃ and the corresponding secondary antibody was incubated for an hour at room temperature. The nuclei were stained by DAPI (Solarbio, Beijing).

### Hoechst33342 staining

COLO 205 and HCT 116 cells treated with coumestrol (100 µM) for 96 hours were fixed with 4% paraformaldehyde in PBS for 20 minutes, and then washed with PBS for three times. Subsequently, Hoechst33342 (#C0031, Solarbio, Beijing) was added and incubated for 5 minutes at room temperature, followed by another three washes with PBS.

### Apoptosis assay

The rate of apoptosis was detected using the Annexin V-FITC/PI apoptosis kit provided by Hangzhou MULTI SCIENCES Co., Ltd. (Hangzhou, China), following the manufacturer's instructions. Briefly, COLO 205 and HCT 116 cells (5×10^5^) treated with coumestrol (100 µM) for 96 hours were collected using EDTA-free trypsin and washed twice with chilled PBS. The cells were then resuspended in 1X binding buffer (500 µL) and stained with 5 µL annexin V-FITC and 10 µL PI staining solution for 5 minutes in the dark at room temperature. Finally, the rate of apoptosis was analyzed by flow cytometry (FACSVerse; BD Biosciences).

### Measurement of iron content

The Ferrous Ion Content Assay Kit (#BC5415) was provided by Beijing Solarbio Science & Technology Co., Ltd (Beijing, China). This kit contains a solution in which ferric ions react with tripyridyltriazine under acidic conditions, resulting in the formation of a blue complex. The content of ferrous ions was subsequently determined by measuring the absorbance value at 593 nm. The detection process strictly adhered to the protocol provided by the manufacturer.

### RNA extraction, reverse transcription, and quantitative real-time PCR

For transcription analysis, total RNA was extracted from cells using RNAiso Plus (Takara) following the manufacturer's instructions. Reverse transcription was performed using the SPARKscript Ⅱ RT Plus Kit (With gDNA Eraser) (#AG0304, SparkJade, China). Quantitative real-time PCR (qPCR) reactions were carried out using 2×SYBR Green qPCR Mix (#AH0101, SparkJade, China). Primer sequences are listed in **Table 1**. Relative expression levels of genes were calculated by ΔΔCt method, which was normalized to β-actin and compared with control samples.

### Western blotting

COLO 205 and HCT 116 cells were lysed using enhanced RIPA lysis buffer (Bioss, Beijing) supplemented with 1 mM PMSF (Solarbio, Beijing). Denatured proteins (30 μg) were separated on 10% SDS-polyacrylamide gels and transferred to PVDF membranes. After blocking with 5% skim milk at room temperature for an hour, the membranes were incubated in primary antibodies overnight at 4 °C. Then, the membranes were washed three times with 0.1% TBST and incubated in secondary antibodies for an hour at room temperature. After being washed three times in 0.1% TBST, the protein signals were detected using an ECL Chemiluminescence Substrate kit (#BL520A, Biosharp, China) on Tanon 6100 system (Shanghai, China). Antibodies used in this study were purchased from ABclonal Technology Co.,Ltd and the detailed information of the antibodies were as follows: NCOA4 Rabbit pAb (A5695), SLC39A8 Rabbit pAb (A22238), VDAC2 Rabbit mAb (A21260), NOX2/gp91phox Rabbit mAb (A19701), β-Actin Rabbit mAb (High Dilution) (AC026), GAPDH Rabbit mAb (A19056), and HRP Goat Anti-Rabbit IgG (H+L) (AS014). The concentration of the antibody dilution was strictly followed as instructed by the manufacturer.

### Small interference RNA (siRNA)

siRNA against the target gene SLC39A8 and negative control siRNA were synthesised by Sangon Biotech (Shanghai) Co., Ltd (Shanghai, China). COLO 205 and HCT 116 cells were transfected with Lipofectamine® RNAiMAX Transfection Reagent (Invitrogen, Carlsbad, California, USA). The target sequences of the SLC39A8 siRNA were as follows 5'-GGTGTGACATGCTATGCAA-3'.

### Statistical analysis

The original data from CCK-8, qRT-PCR, WB, ferrous ion content detection, and flow cytometry analysis of cell apoptosis were analyzed using GraphPad Prism software (GraphPad Software, San Diego, CA, USA). Image J software was employed to measure the grayscale values of WB protein bands. Flowjo software was used to process the experimental image data for flow cytometry analysis of cell apoptosis. Significance analysis was conducted using t-test for overall comparison, and a difference was deemed statistically significant at *P<0.05*.

## Results

### SLC39A8 was a tumor associated gene of CRC

**Figure 1:**
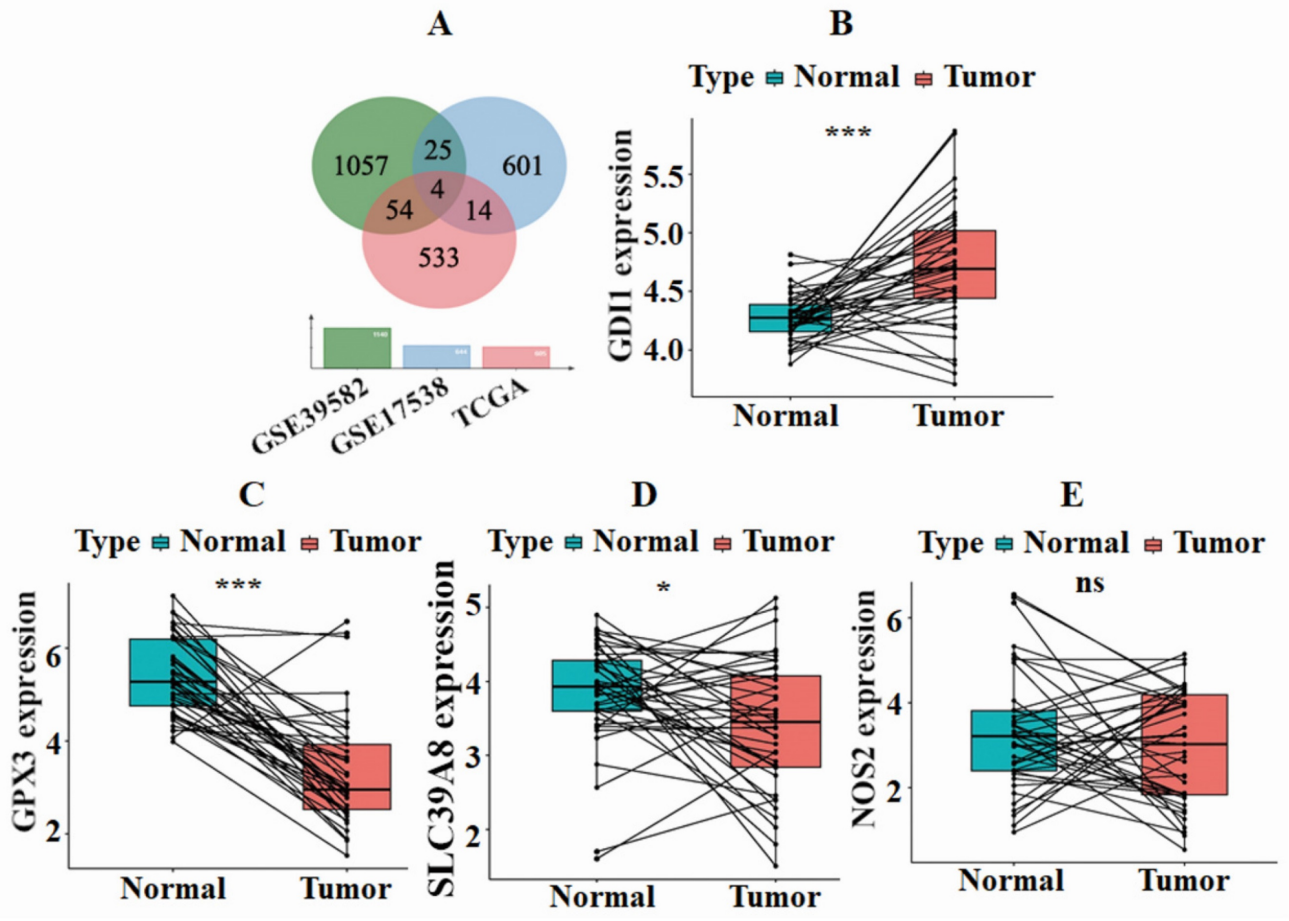
** SLC39A8 was associated with survival of CRC**. Wayne's analysis revealed that four genes intersected across the three datasets: TCGA, GSE39582, and GSE17538 (A). In paired differential analysis, GDI1 demonstrated significant up-regulation, while GPX3 and SLC39A8 were down-regulated in tumor samples compared with normal samples, and no significant difference was observed in NOS2 (B-E).

To identify genes that are significantly associated with survival, a single-factor COX analysis was conducted. For GSE39582, GSE17538, and TCGA-COAD datasets, 3127, 3117, and 1297 prognosis-related genes were screened (data not shown). To analyze the correlation between genes and patient survival time, survival status, and tumor TNM, as well as to identify genes that differ between different clinical characteristics, clinical correlation analysis was conducted. The number of prognosis-related genes with significant expression differences was 1140 in GSE39582, 644 in GSE17538, and 605 in TCGA-COAD. By intersecting the clinical correlation genes from the three datasets, we identified a total of four common genes: GDI1, GPX3, NOS2, and SLC39A8. These results are visually represented in** Fig. 1A**.

To investigate the expression of intersection genes in both normal and tumor samples from the same patient, we performed a paired differential analysis. The results, depicted in **Fig. 1B-E**, revealed significant differences in the expression levels of GDI1, GPX3, and SLC39A8 between normal and tumor samples (*P<0.05*). However, no noteworthy difference was observed in NOS2. Moreover, the expression of GDI1 was up-regulated in tumor samples, whereas GPX3 and SLC39A8 were down-regulated in tumor samples.

**Figure 2:**
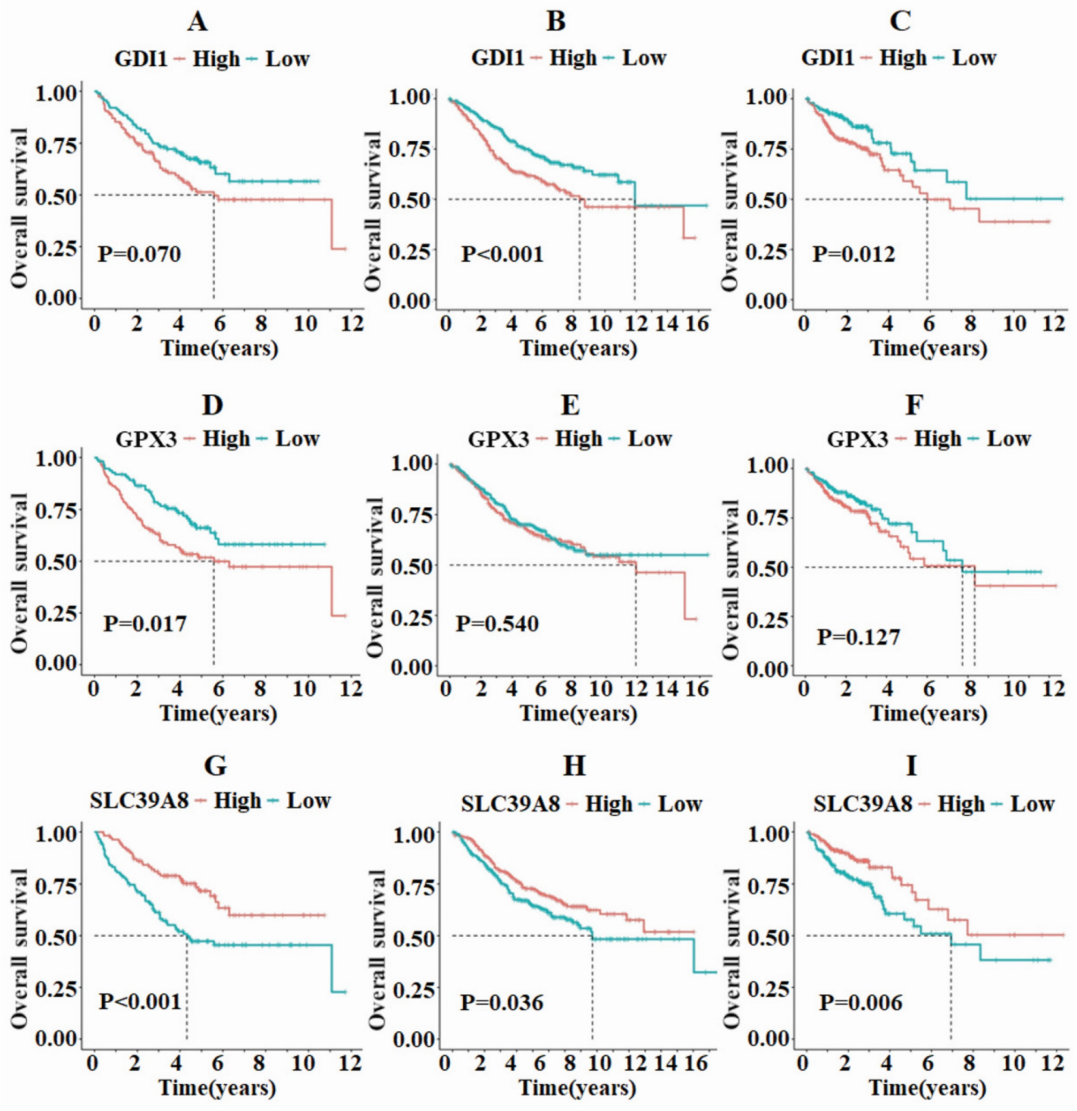
The survival analysis of GDI1, GPX3, and SLC39A8 in the three datasets TCGA, GSE39582, and GSE17538 revealed that SLC39A8 displayed distinct survival outcomes between the high and low expression groups.

Survival analysis was then conducted on the three differentially expressed genes to examine the relationship between death rate, survival rate, and survival time. In the GSE17538 and TCGA-COAD datasets, GDI1 exhibited different survival statuses between the high and low expression groups, while no such difference was observed in the GSE39582 dataset (**Fig. 2A-C**). On the other hand, GPX3 only showed differing survival statuses in the high and low expression groups in the GSE17538 dataset, but not in the GSE39582 and TCGA-COAD datasets (**Fig. 2D-F**). In all three datasets, SLC39A8 demonstrated varying survival analysis outcomes, with the high expression group displaying a better prognosis compared to the low expression group (**Fig. 2G-I**).

**Figure 3:**
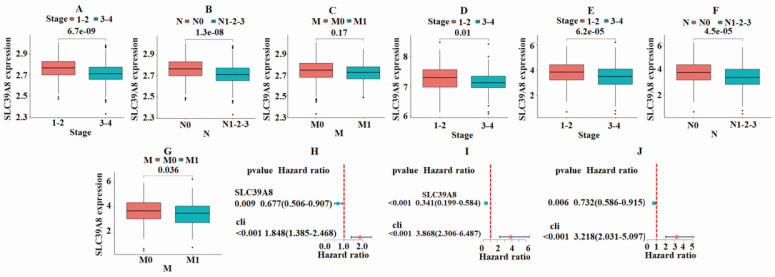
** Results of correlation analysis between the expression of SLC39A8 and clinical classification indicators.** In the GSE39582 dataset, a significant difference was noted in the expression of SLC39A8 among patients with different stages (A) and N-stages (B), but not M-stage (C). The GSE17538 dataset indicated significant variations (*P*<0.05) in the expression of SLC39A8 between patients at early and late stages (D). In the TCGA dataset, a significant disparity was observed in the expression of SLC39A8 across patients with varying stages (E), N-stages (F), and M-stage (G). Single factor independent prognostic analysis showed that SLC39A8 could serve as an independent prognostic factor for CRC (H-J).

To further analyze the role of tumor-associated gene in CRC, a correlation analysis was conducted between the expression of SLC39A8 and clinical classification indicators. As shown in **Fig. 3A**, the GSE39582 dataset demonstrated a significant difference (*P*<0.05) in the expression of SLC39A8 between patients in early and late stages of cancer. The expression was higher in early-stage patients compared with late-stage patients. The results also showed significant differences (*P<0.05*) based on the N-stage, indicating the presence or absence of lymph node metastasis (**Fig. 3B**). These differences were more pronounced in patients without metastasis, where the expression of SLC39A8 was higher than in those with metastasis. In contrast, the expression of SLC39A8 was not significantly related to M-stage (**Fig. 3C**). Similarly, the GSE17538 dataset revealed significant differences (*P*<0.05) in SLC39A8 expression between early and late stage patients, with higher expression in early-stage cases (**Fig. 3D**). However, due to a lack of information on N-stage and M-stage status, a comparison could not be made in this dataset. In the TCGA-COAD dataset, the results were consistent with those of the GSE39582 dataset, showing significant differences (*P*<0.05) in SLC39A8 expression based on pathological staging and N-stage. However, a significant difference (P=0.036) was found in M-stage. Additionally, the expression of SLC39A8 was found to be significantly different in relation to N-stage, with higher expression observed in patients without distant metastasis. Overall, the findings suggest that the expression of SLC39A8 may be a useful predictor of cancer progression, particularly in relation to N-stage evaluation. Further research is necessary to investigate its potential application in clinical settings. Single-factor independent prognostic analysis demonstrated that SLC39A8 can serve as an independent prognostic factor for CRC across three datasets (**Fig. 3H-J**). Taken together, these results indicate that SLC39A8 could be a potential gene associated with CRC clinical progression staging.

### Coumestrol is the potential monomer that acts on SLC39A8 encoding protein

**Figure 4:**
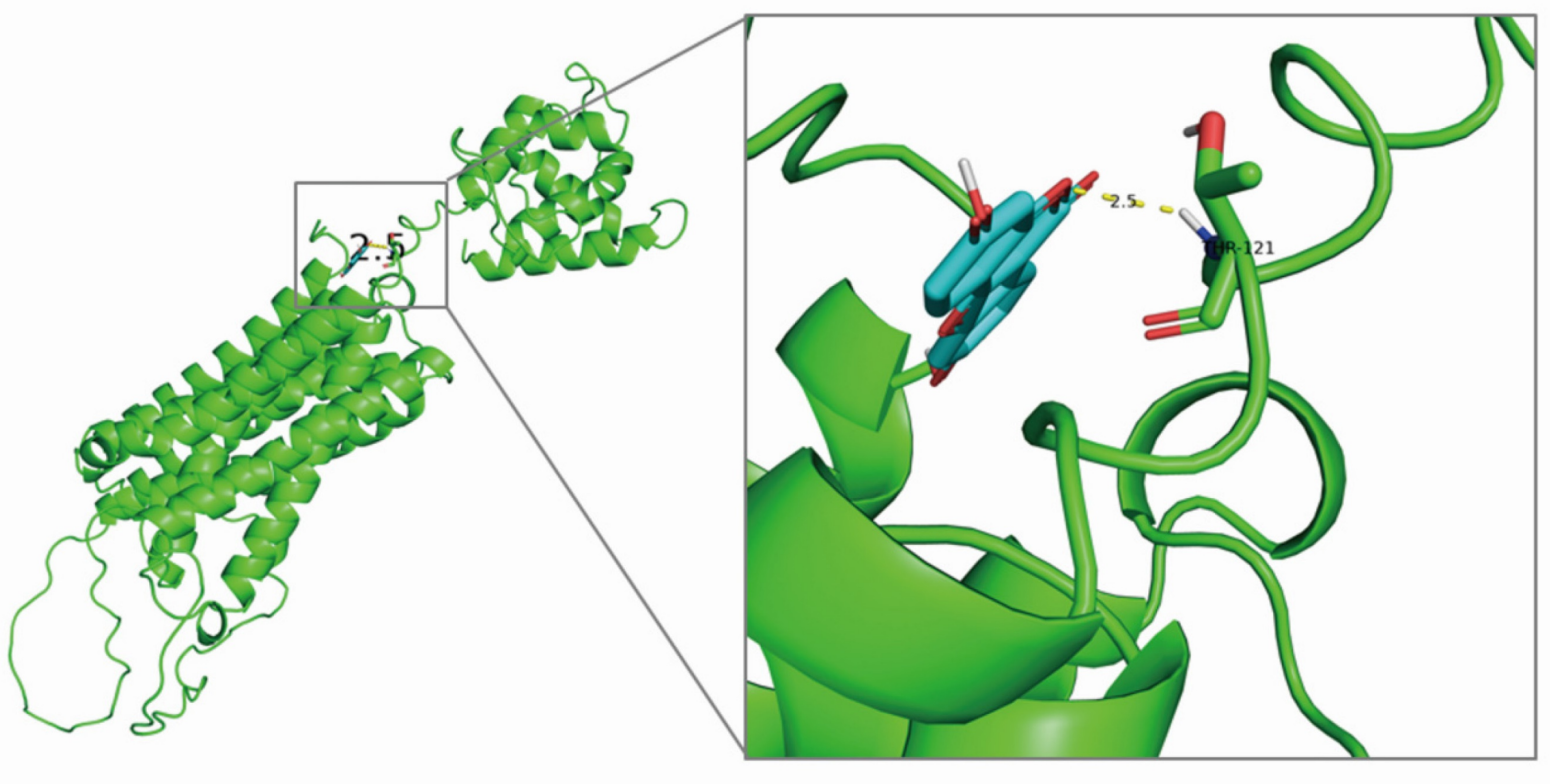
** Structural modeling revealed that coumestrol and ZIP8 could form a hydrogen bond of 2.5** Å**.**

SLC39A8 encodes ZIP8, a highly efficient transport protein responsible for the movement of divalent iron and manganese 13. To identify potential monomers that interact with ZIP8, a screening using the HERB and TCMSP databases was conducted. The HERB database retrieved four components: 17-alpha-estradiol, 17-beta-estradiol, 17-beta-oestradiol, and coumestrol. After screening with the TCMSP database, only coumestrol (OB=32.49%, DL=0.34) met the requirements. Coumestrol can be obtained from various Chinese herbal medicines, such as jujube, kudzu flower, and kudzu root. Therefore, coumestrol was selected as the candidate traditional Chinese medicine component for this study. To investigate the potential interaction between coumestrol and ZIP8, molecular docking analysis was conducted. The resulting binding diagram is shown in** Fig. 4.** Our findings revealed that coumestrol forms a hydrogen bond of 2.5Å with THR-121 of ZIP8, enabling effective binding of coumestrol to ZIP8.

### ZIP8 may play a significant role in CRC development by ferroptosis pathway

**Figure 5:**
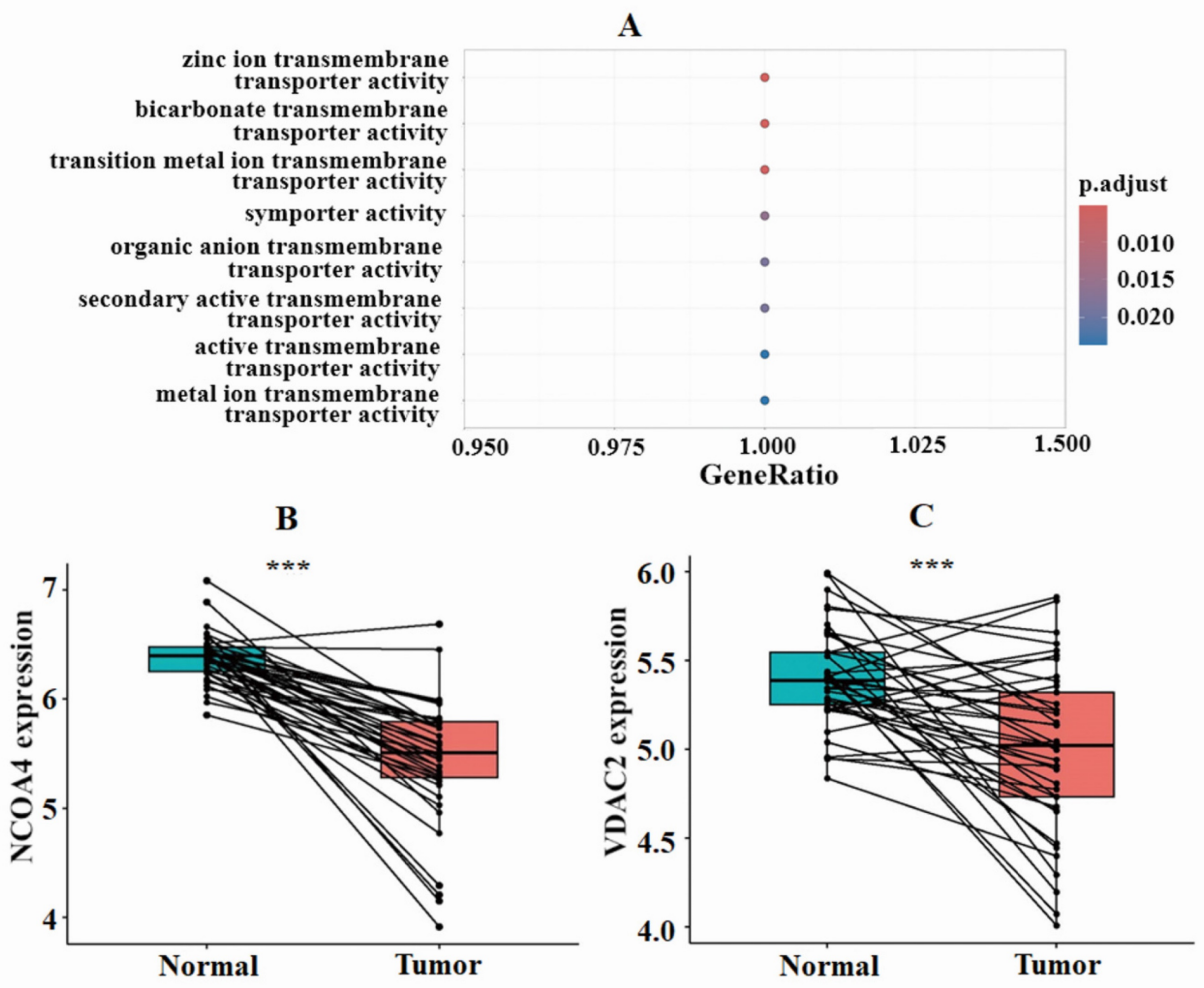
** Functional enrichment analysis indicated the involvement of ferroptosis pathway-related genes in CRC development.** GO analysis indicated that ZIP8 primarily functions as a transporter (A). Paired differential analysis of ferroptosis pathway-related genes NCOA4 and VDAC2 using TCGA data revealed significant differences between high and low groups.

To gain a better understanding of how ZIP8 contributes to the CRC cell signaling pathway, we conducted GO and KEGG enrichment analyses. The results of the GO analysis indicated that ZIP8 primarily functions as a transporter, specifically in activities such as zinc ion transmembrane transport, bicarbonate transmembrane transport, and transition metal ion transmembrane transport (**Fig. 5A**). ZIP8 also regulates molecular functions such as iron, manganese, and zinc ion transmembrane transporter activities. The KEGG analysis revealed that ZIP8 may play a significant role in CRC development through the ferroptosis pathway (**Supplementary Fig. 1**). This pathway involves three additional target proteins, namely, NCOA4, VDAC2, and NOX2, which could potentially be affected by ZIP8. Paired differential analysis using TCGA dataset revealed significant differences in the expression levels of NCOA4 and VDAC2 between CRC tissue and normal tissue (**Fig. 5BC**). Interestingly, these genes were found to be down-regulated in tumor tissue. It should be noted that NOX2 expression was not included in TCGA dataset.

### Coumestrol affects CRC cell proliferation and apoptosis

**Figure 6:**
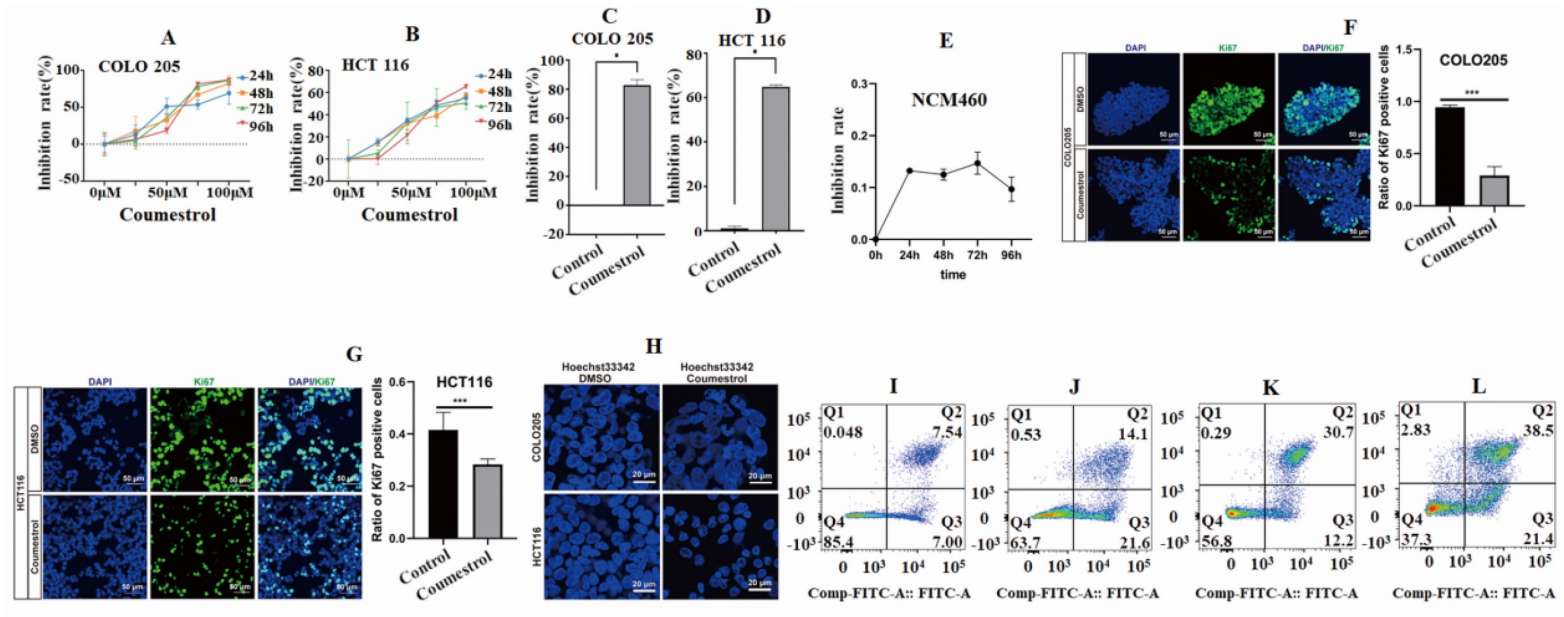
** Cell inhibition and apoptosis analysis results.** Drug concentration screening using CCK-8 showed better cell inhibition rates of 100 µM coumestrol for 96 hours (A-D). The CCK-8 results show that coumestrol had a weaker inhibitory effect on the normal cell line NCM460 (E). Immunofluorescence staining demonstrates that coumestrol can inhibit the proliferation of CRC cell lines (FG). Immunofluorescence staining suggests that coumestrol could promote apoptosis in CRC cell lines (H). Increased cell apoptosis was noted in both COLO 205 and HCT 116 cells in coumestrol group while compared with the DMSO control group (I-J), ordinate: Comp-Propidium lodide-A:: Propidium lodide-A.

To investigate the effects of coumestrol on CRC, a cellular experiment was conducted using COLO 205 and HCT 116 cell lines. The cell inhibition rates were observed in both cell lines within the range of 0-100 µM after applying coumestrol for 24, 48, 72, and 96 hours. This was determined using the CCK8 assay and compared to the control group. It was noted that the cell inhibition rates of both cell lines increased gradually, with the most significant effect seen at 100 µM (**Fig. 6AB**). Therefore, the concentration and duration of coumestrol were set at 100 µM for 96 hours. **Fig. 6CD** showed that the coumestrol group exhibited approximately 82.74% and 64.81% increase in cell inhibition rates for COLO 205 and HCT 116 cell lines, respectively, compared with the DMSO control group (*P*<0.05). To test whether coumestrol also exhibits strong inhibitory effects on normal intestinal cells, coumestrol was added to the NCM460 cell line and a CCK-8 assay was performed. The results showed that the inhibitory effect of coumestrol on the NCM460 cell line was much lower than that on the CRC cell lines (**Fig. 6E**). To test whether coumestrol can affect CRC cell proliferation, immunofluorescence staining experiment for the proliferative associated gene Ki67 was also performed. The results demonstrated that the coumestrol groups had significantly fewer Ki67-positive cells than the DMSO cotrol groups (**Fig. 6FG**). To investigate the impact of coumestrol on the apoptosis of CRC cells, Hoechst 33342 staining and flow cytometry detection were performed. The cells treated with coumestrol displayed more fragmented, condensed, and less clearly defined nuclei compared with the DMSO control groups, indicating a higher presence of apoptotic or necrotic cells (**Fig. 6H**). The group treated with 100 µM coumestrol showed a substantial increase in apoptosis in COLO 205 cells compared with the DMSO control group, as shown in **Fig. 6IJ**. The late-stage and early-stage apoptosis in COLO 205 cells recorded a rise of 138% and 162% respectively. Similarly, HCT 116 cells also exhibited a significant increase in apoptosis after treatment with 100 µM coumestrol, with late-stage and early-stage apoptosis increasing by 29% and 21% (**Fig. 6KL**), respectively. These findings provide compelling evidence that coumestrol plays a crucial role in inducing proliferation inhibition and apoptosis in CRC cell lines.

### Coumestrol induces the expression of genes involved in the ferroptosis pathway in CRC cells

**Figure 7:**
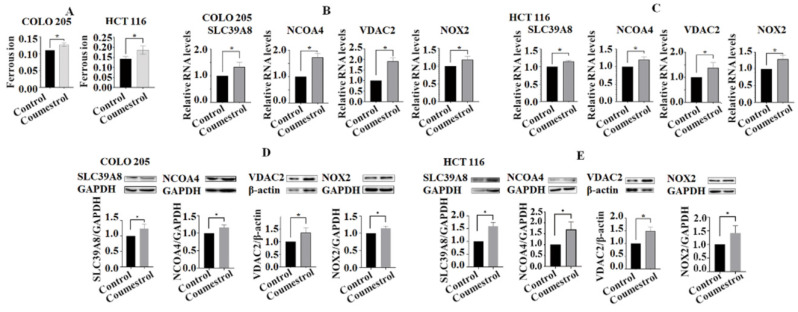
** Effects of coumestrol on the expression of ferroptosis pathway genes in CRC** cells. Ferrous ions increased in both COLO 205 and HCT 116 cells after coumestrol treatment (A). The RT-PCR (B-C) and Western blot (D-E) analyses revealed that there was an increase in the expression of ferroptosis pathway-related genes, namely SLC39A8, NCOA4, VDAC2, and NOX2, at both the mRNA and protein levels following treatment with coumestrol.

To investigate the potential impact of coumestrol on the ferroptosis pathway, the levels of ferrous ions in cell lines after exposure to coumestrol were evaluated. The results demonstrated a significant increase in the content of ferrous ions in both COLO 205 and HCT 116 cells (**Fig. 7A**). Additionally, RT-PCR and Western blot analyses were performed to assess the expression of key genes involved in the ferroptosis pathway, namely SLC39A8, NCOA4, VDAC2, and NOX2, in CRC cells treated with coumestrol. Our findings revealed a noticeable upregulation of all four genes in COLO 205 cells, and a similar trend was observed in HCT 116 cells after treatment at both the mRNA (**Fig. 7B-C**) and protein (**Fig. 7D-E**) levels. Taken together, these results indicate that coumestrol induces the expression of genes involved in the ferroptosis pathway in CRC cells.

### Coumestrol affects ferroptosis pathway in CRC cell lines by directly interacting with ZIP8

**Figure 8:**
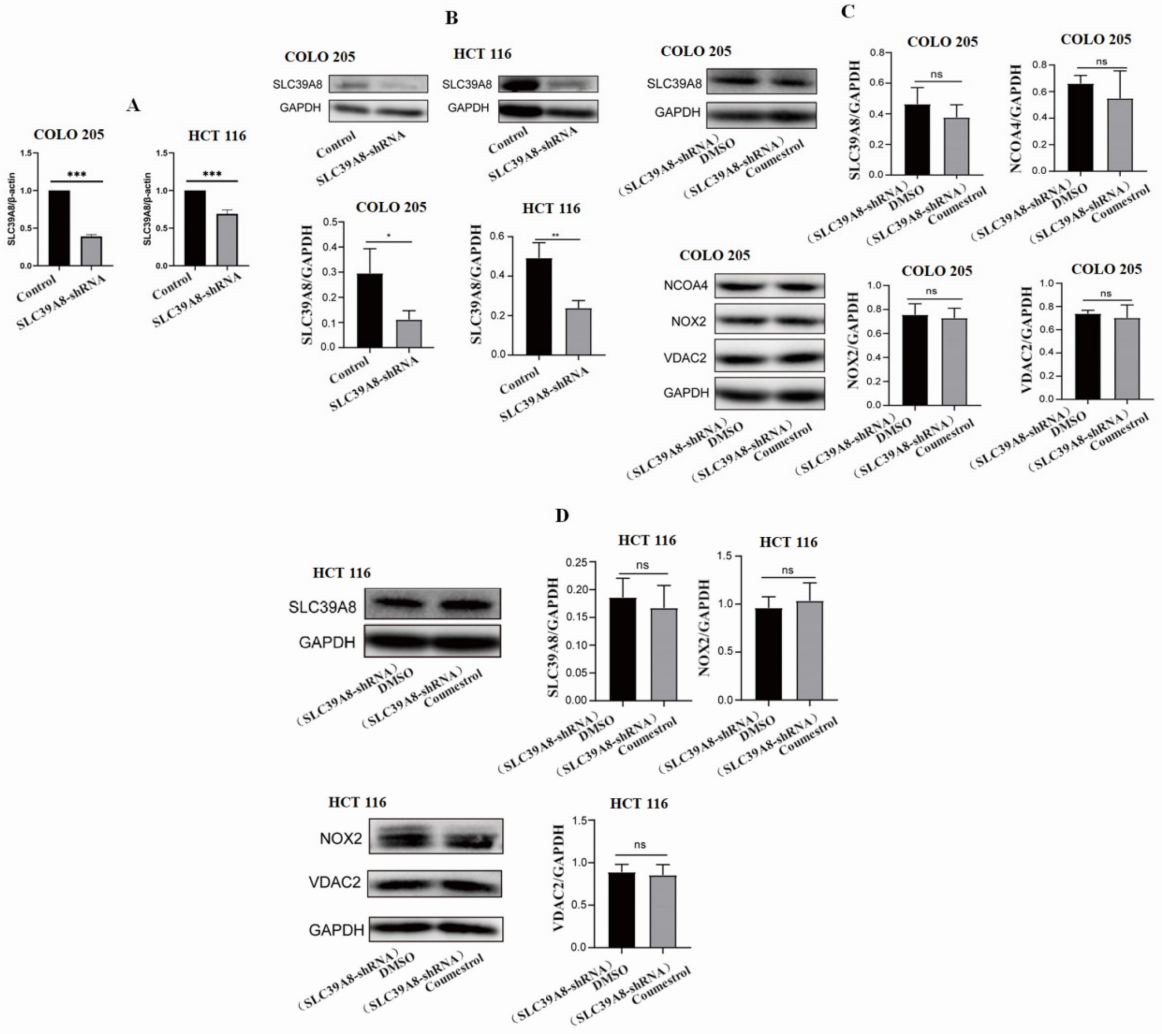
** Coumestrol affects ferroptosis pathway in CRC cell lines.** SLC39A8 expression decreased at both mRNA (A) and protein levels (B) in both COLO 205 and HCT 116 cell lines after 96 hours of siRNA interference. Coumestrol could not upregulate ferroptosis pathway-related genes in CRC cell lines treated with SLC39A8 interference (C-D).

In this study, our structural modeling indicated a direct interaction between ZIP8 and coumestrol. The results from RT-PCR and WB experiments also supported this hypothesis. To further verify the direct interaction between ZIP8 and coumestrol, we conducted siRNA interference and examined the expression of SLC39A8 and other ferroptosis pathway-related genes. **Fig. 8AB** displayed a significant decrease in the expression of SLC39A8 at both mRNA and protein levels in both COLO 205 and HCT 116 cell lines after 96 hours of siRNA interference, indicating successful interference. **Fig. 8CD** demonstrated that after 72 hours of interference, there was no significant increase in the expression of SLC39A8 and other ferroptosis pathway-related genes following the administration of coumestrol. Since the administration of coumestrol after interference does not rescue the expression levels of the SLC39A8-related pathway, our results indicate that coumestrol affects the ferroptosis pathway in CRC cell lines by directly interacting with ZIP8.

## Discussion

Analysis of a single dataset or database might lead to unverifiable results due to the heterogeneity of the data being analyzed. To address this issue, we chose to perform a cross-analysis of data from multiple sources, namely GSE39582, GSE17538, and TCGA-COAD, as they provide sufficient clinical information. By performing clinical correlation analysis, we conducted a preliminary screening of four target genes: GDI1, GPX3, NOS2, and SLC39A8. These genes have been reported to play important roles in cancer development 14-17. For instance, the methylation state of GPX3 has been identified as a potential predictor of platinum sensitivity in CRC 15. Further analysis revealed that only SLC39A8 showed significant significance in all three datasets, leading us to designate it as the core prognostic gene in this study. Notably, SLC39A8 exhibited significant expression differences in cases of lymph node metastasis and distant metastasis in CRC patients. Interestingly, a study by Liu *et al*. demonstrated that SLC39A8 also plays a role in the migration and invasion of renal cancer 18. Our independent prognostic analysis further revealed an association between SLC39A8 and the prognosis of CRC patients, which aligns with the findings of Lius' study on renal cell carcinoma 18. Moreover, SLC39A8 has also been considered a potential prognostic target in esophageal squamous cell carcinoma 19. Hence, the expression of SLC39A8 appears to be associated with the development of various tumors and holds potential clinical value.

The combination of traditional Chinese medicine monomers with molecular targets has made traditional Chinese medicine treatment and new drug development increasingly popular, leading to the emergence of a variety of cancer treatment methods and combination therapy strategies. Han *et al.* utilized network pharmacology to identify key targets of curcumin for the treatment of colorectal cancer, including AKT1, EGFR, and STAT3 20. Wang et al. demonstrate that the ethanol extract of Retinervus Luffae Fructus, a traditional Chinese medicine, can inhibit the growth of mouse CRC cells and the proliferation of SW480 cells through the Wnt/β-Catenin pathway 21. Different from conventional methods in network pharmacology 22, this study employed a progressive approach to screen the active ingredients of traditional Chinese medicine. Initially, disease targets were identified, followed by the gradual screening of corresponding ingredients. This approach enabled us to investigate the effects of a single traditional Chinese medicine ingredient on a specific disease target, successfully avoiding any uncertainties that may arise from multiple ingredient mechanisms. Through our screening process, it was revealed that coumestrol has the potential to bind to the protein ZIP8, which is encoded by the gene SLC39A8. SLC39A8 plays a role in the cellular response to the anti-cancer drug cisplatin 23. Additionally, Kim *et al*. found that coumarine has a broad inhibitory effect on the growth of melanoma, lung cancer, and colorectal cancer cells. Through GO and KEGG analysis, this study established a significant association between the SLC39A8 gene, the activity of the iron-ion transmembrane transporter, and the ferroptosis pathway. Zafar *et al*. propose that the metal copper can interact with coumestrol, leading to ROS-mediated DNA damage and cell death 25. We have formulated a hypothesis that coumestrol may function by interacting with ZIP8 to facilitate the ferroptosis pathway. This, in turn, may result in apoptosis of CRC cells and ultimately contribute to a therapeutic effect on tumors.

Due to limited references in the literature regarding the administration concentration of coumestrol, the CCK-8 method was employed as the first step to screen the administration concentration. The findings revealed that coumestrol impeded the growth of CRC cells in a dosage-dependent manner. The maximum inhibition rate of CRC cells was observed 96 hours after the administration of 100 µM coumestrol, without any noticeable signs of cell toxicity. In a study conducted by Kim et al., they also observed a dose-dependent anticancer activity of coumestrol in HT 29 and HCT 116 CRC cells 24, which is consistent with our own results.

Iron ions, an essential trace element, participate in cell proliferation, metabolism, and differentiation. The occurrence and development of CRC heavily rely on iron metabolism 17. SLC39A14 has the capability to facilitate the entry of zinc, manganese, iron, cadmium, and cobalt, thus highlighting the significance of its expression for divalent iron ions, which play a vital role in cell death 26, 27. SLC39A8 belongs to the SLC39 transporter protein family and might have similar functions to SLC39A14. Ferroptosis plays a crucial role in suppressing tumors and can be utilized for cancer treatment 28. Our results showed that after the administration of coumestrol, the intracellular ferric ion content in CRC cells increases, suggesting an increase in iron metabolism in cancer cells. Consistently, it has been mentioned that SLC39A8, due to its capability to transport selenium, has the potential to enhance the effectiveness of anti-cancer drugs and could serve as a novel target for cancer treatment 29. Additionally, other researchers have discovered that coumestrol can inhibit CRC cells through alternative pathways. Coumestrol is capable of quinone reductase induction in Colo205 cells by promoting quinone reductase mRNA expression 30. Javid et al. demonstrated that coumestrol prevents intestinal tumorigenesis and improves enterocyte migration and intercellular adhesion in the Apc(Min/+) mouse model of colorectal cancer 31. Lee et al. discovered that coumestrol induces senescence in human breast cancer and CRC cells by inhibiting protein kinase CKII, resulting in the production of reactive oxygen species 32. Based on our findings and previously reported research results, we believe that the anticancer mechanism of coumestrol is likely to be complex, and therefore requires further extensive research to elucidate its mechanisms.

In both COLO 205 cells and HCT 116 cell lines, cell proliferation was inhibited after treatment with coumestrol. Additionally, the expression of the SLC39A8 gene was up-regulated at both the mRNA and protein levels. Furthermore, the expression of the NCOA4, VDAC2, and NOX2 genes in the ferroptosis pathway also increased, suggesting that these genes may be altered after treatment with SLC39A8. Another study discovered that the over expression of SLC39A8 effectively suppressed the proliferation rate, migration, and invasion of renal cancer cells, which is consistent with the findings of this study 18. After conducting SLC39A8 gene interference on two cell lines, we found that, with the administration of coumestrol, the up-regulation of ferroptosis pathway-related genes was not possible. This implies that there is a direct interaction between coumestrol and SLC39A8, rather than just a correlation.

## Conclusion

In summary, by integrating bioinformatics analysis with relevant cellular experiments, we have generated preliminary data that supports the potential use of coumestrol as an adjuvant therapy for CRC. However, it should be noted that this study does have certain limitations. We only investigated the effects of coumestrol on CRC at the cellular level. In reality, the drug metabolism within the body is far more complex than what can be studied through simple cell experiments. Hence, in the future, we intend to conduct tests to examine the in vivo effects of coumestrol.

## Supplementary Material

Supplementary figure.

## Figures and Tables

**Table 1:** Primer sequencing used in this study.

Primer name	Sequences (5'-3')	Amplification length
NCOA4-FP	CCGTTGGGAATTTTCAGATCC	75bp
NCOA4-RP	TGTCCATTCCTTCACATCTTCG	75bp
NOX2-FP	GGCTGGGGTTGAACGTCTT	104bp
NOX2-RP	CAGTGCCAGTGCTGACCCAA	104bp
SLC39A8-FP	CCCCACGAGTTAGGAGACTT	115bp
SLC39A8-RP	TGCCAAAAGCTAGCCCAACA	115bp
VDAC2-FP	GAGCTCAGATTGCCTGCCCTT	147bp
VDAC2-RP	TTTCACCAACCCAAAACCAAATCC	147bp
β-actinFP	CTCCATCCTGGCCTCGCTGT	268bp
β-actinRP	GCTGTCACCTTCACCGTTCC	268bp
