# Supplementary material for: Coumestrol facilitates apoptosis in colorectal cancer cells by interacting with ZIP8 protein via the ferroptosis pathway
Source: J Cancer. 2024 Jul 2;15(14):4656–67. doi: 10.7150/jca.94628 (PMC11242349; doi:10.7150/jca.94628)
Supplement: Supplementary file 1 — Supplementary figure. [file jcav15p4656s1.pdf]

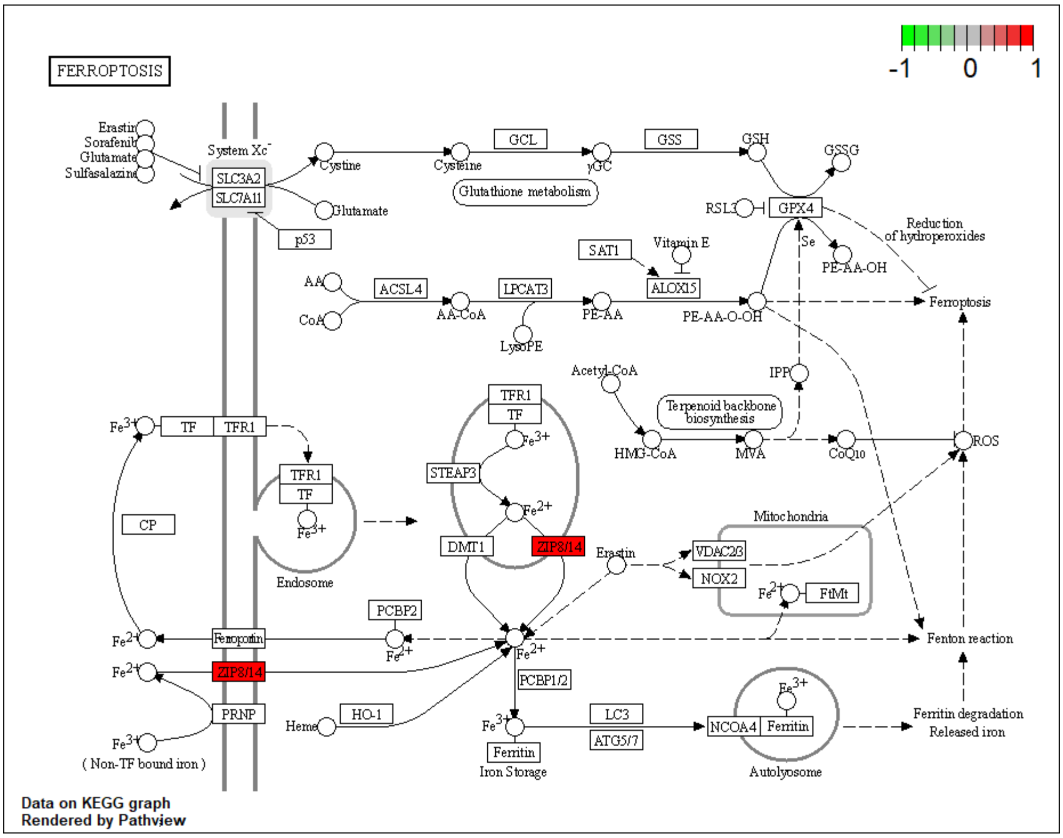

Supplementary Fig.1 KEGG functional enrichment analysis showed the involvement of ferroptosis pathway-related genes SLC39A8, NCOA4, VDAC2, and NOX2 in CRC development.
